# Constructing a signature based on the SIRT family to help the prognosis and treatment sensitivity in glioma patients

**DOI:** 10.3389/fgene.2022.1035368

**Published:** 2022-12-09

**Authors:** Feiyue Xuan, Zhiwei Zhang, Kuili Liu, Haidong Gong, Shaodong Liang, Youzhi Zhao, Hongzhe Li

**Affiliations:** ^1^ Department of Neurosurgery, Hongqi Hospital Affiliated to Mudanjiang Medical University, Mudanjiang, China; ^2^ Heilongjiang Provincial Key Laboratory of Cancer Disease Prevention and Control, Mudanjiang Medical University, Mudanjiang, China

**Keywords:** glioma, SIRT family, biomarker, prognosis, tumour microenvironment

## Abstract

Enzymes of the silent information regulator (SIRT) family exert crucial roles in basic cellular physiological processes including apoptosis, metabolism, ageing, and cell cycle progression. They critically contribute to promoting or inhibiting cancers such as glioma. In the present study, a new gene signature of this family was identified for use in risk assessment and stratification of glioma patients. To this end, the transcriptome and relevant clinical records of patients diagnosed with glioma were obtained from the Cancer Genomic Atlas (TCGA) and the Chinese Glioma Genome Atlas (CGGA). LASSO regression and multivariate Cox analyses were used to establish the signature. Using Kaplan–Meier analyses, overall survival (OS) was assessed and compared between a training and an external test datasets which showed lower OS in patients with high risk of glioma compared to those with low risk. Further, ROC curve analyses indicated that the SIRT-based signature had the desired accuracy and universality for evaluating the prognosis of glioma patients. Using univariate and multivariate Cox regression analyses, the SIRT-based signature was confirmed as an independent prognostic factor applicable to subjects in the TCGA and CGGA databases. We also developed an OS nomogram including gender, age, risk score, pathological grade, and IDH status for clinical decision-making purposes. ssGSEA analysis showed a higher score for various immune subgroups (e.g., CD8^+^ T cells, DC, and TIL) in samples from high-risk patients, compared to those of low-risk ones. qPCR and western blotting confirmed the dysregulated expression of SIRTs in gliomas. Taken together, we developed a new signature on the basis of five SIRT family genes, which can help accurately predict OS of glioma patients. In addition, the findings of the present study suggest that this characteristic is associated with differences in immune status and infiltration levels of various immune cells in the tumor microenvironment.

## Introduction

Among the various brain tumors, glioma is the most frequent malignancy, which occurs in adults and accounts for almost one-third of primary brain tumors in people aged 20 59 (Jiang et al., 2016). Because of its extensive proliferation, invasion, immunosuppression, and resistance to chemotherapy, clinical treatment of glioma is notoriously difficult (Jiang et al., 2021; Qi et al., 2022). The median survival time of patients diagnosed with low-grade glioma is approximately 6 years. Compared with low-grade gliomas (LGG, WHO grades II and III), glioblastoma (GBM, WHO grade IV) is more often fatal (Ostrom et al., 2021). The median survival time in glioblastoma is typically less than 2 years, even when implementing large-scale surgery, focal resection, and combined radiotherapy and chemotherapy (Linz, 2009). Thus, there is an urgent need for new biomarkers to facilitate accurate diagnosis and to improve the currently limited therapeutic options.

Silent information regulators (SIRTs), or NAD-dependent deacetylase sirtuins, are class-III histone deacetylases responsible for the unique β-nicotinamide adenine dinucleotide (β-NAD+)-dependent Nε-acyl-lysine deacylation of both histone and non-histone substrates (Teixeira et al., 2020). Sirtuin proteins are highly conserved and occur as seven subtypes in humans, i.e., SIRT1 to SIRT7 (Teixeira et al., 2020). Sirtuins contribute to the regulation of various biological processes, including apoptosis, metabolism, ageing, and cell cycle progression (Brown et al., 2013; Li et al., 2013). Given that, sirtuins are considered to be closely associated with the onset and progression of tumors (Brown et al., 2013; Li et al., 2013), and increasing evidence confirms the importance of the SIRT family in glioma, as these proteins may promote or inhibit cancer progression (Chen et al., 2018; Funato et al., 2018; Luo et al., 2018; Mu et al., 2019; Yalçın and Colak, 2020; Liu et al., 2022). However, most previous studies focused on exploring the biological functions of SIRT family members in gliomas, whereas their prognostic value for glioma patients has been overlooked.

Using comprehensive bioinformatics, we performed data-mining through transcriptional and clinical records of patients diagnosed with glioma using two databases procured from the Cancer Genomic Atlas (TCGA) and the Chinese Glioma Genome Atlas (CGGA), respectively. We further investigated the role of SIRT family members in the diagnosis, prognosis, and treatment of gliomas through clinical sample validation. We present the following article in accordance with the TRIPOD reporting checklist.

## Materials and methods

### Data collection

The sequenced transcriptome and clinical data of 689 patients diagnosed with glioma were downloaded from the TCGA-GBM and TCGA-LGG projects using the UCSC Xena database (http://www.genome.ucsc.edu/index.html). Further, normal brain tissue samples composed of paracancerous tissues in TCGA-GBM (n = 5) and GTEX (n = 1,152) were retrieved from the UCSC Xena database. The retrieved transcriptome records were converted to TPM format for subsequent analysis as a training data set. For subsequent validation, the transcriptome and clinical information of the validation dataset (CGGA, n = 325) were also downloaded from the CGGA database (http://www.cgga.org.cn/index.jsp).

From September 2021 to March 2022, three cases of glioma tissues and their relative paracancerous tissue were extracted from clinical subjects who were schedules to receive initial surgery in Hongqi Hospital Affiliated with Mudanjiang Medical College. This project was performed under the approval of the medical ethics committee of Hongqi Hospital Affiliated with Mudanjiang Medical College. All participants signed written informed consent. Information about the participants is provided in Supplement 1.

### Differential analysis of the SIRT family in gliomas

We first used high-throughput sequencing data from TCGA and GTEx to investigate the gene expression levels of SIRT family members in the glioma samples and the respective adjacent normal brain tissues. Subsequently, differential expression of SIRT family genes was compared between the glioma samples and adjacent normal brain tissues, across the Human Protein Atlas (HPA; https://www.proteinatlas.org). Then, the potential of this gene family for differentiating glioma tissues from normal brain tissues was evaluated using ROC curves. Higher AUC values indicated better differentiation ability.

### cBioPortal database

The open-source database cBioPortal (http://www.cbioportal.org) is designed for exploring the genetic changes of a gene in cancer, including its mutations, copy number variation, and relationship with clinical features (Gao et al., 2013). cBioPortal was thus used to assess the genetic changes of SIRT genes and their prognostic value in glioma patients.

### Survival analyses

The prognostic indicators of this study included overall survival (OS), disease-specific survival (DSS), and the progression-free interval (PFI). SIRT genes were categorized as either highly expressed (HE) or low expressed (LE), according to their median mRNA abundance. The prognostic differences between the HE and LE groups were compared by Kaplan–Meier survival analyses. Univariate and multivariate Cox analyses were performed to evaluate the potential of SIRT family genes as independent prognostic factors in patients diagnosed with glioma.

### Protein-protein interaction (PPI) network

GeneMANIA database (http://genemania.org) was used for exploring the functions and interactions between the targeted genes (Franz et al., 2018). We explored the proteins interacting with SIRT genes in humans and their functions and used them to construct a PPI network. GeneMANIA is an online database that contains 660,554,667 interactions and 166,691 genes of nine species.

### Functional enrichment analysis

The Metascape online tool (https://metascape.org/gp/index.html#/main/step1) provides comprehensive gene annotations (Zhou et al., 2019). In this study, we used Metascape to perform functional enrichment analysis and visualization of the SIRT family and its interacting genes. The “clusterProfiler” package in R software version 4.1.1 was applied for gene set enrichment analyses (GSEA) using the C2 KEGG database, with *p* < 0.05 and *q* < 0.25 considered significant.

### Constructing a SIRT-based signature

To create a signature based on the SIRT family in cases with glioma, the TCGA queue was used as a train dataset. In the train dataset, SIRT family genes were subjected to LASSO regression analysis using the R software packages “glmnet” and “survival”, and overfitted genes were excluded. Multivariate Cox regression analysis was used to determine the most significantly and valuable prognostic genes in the SIRT family. Then, the corresponding regression coefficients and risk scores of glioma patients were calculated using the following equation:

$$Risk\;score=SIRT1\times \left(-0.961\right)+SIRT2\times \left(-0.318\right)+SIRT5\times \left(-0.473\right)+SIRT6\times \left(-0.239\right)+SIRT7\times \left(0.784\right)$$

Subsequently, the CGGA data were used as a test dataset to determine the risk score of patients diagnosed with glioma in the CGGA database. Subjects were further stratified in high-risk (HR) and low-risk (LR) groups, depending on their median risk scores. Kaplan–Meier survival analysis was applied to assess the prognosis difference between the patients form groups with high risk and low risk of glioma. The performance of the SIRT-based signature was evaluated using a ROC curve, and the independent prediction ability of the SIRT-based signature was determined through univariate and multivariate Cox analyses.

### Immune infiltration analysis

According to the transcriptome data, infiltration of various immune cells and immune status scores of glioma patients in the TCGA data set were evaluated using a single-sample GSEA (ssGSEA) algorithm. Then, the immune cell content and immune status scores were compared between patients of the HR and LR groups.

### Drug sensitivity analysis

Microsatellite installation (MSI) and tumor immune dysfunction and exclusion (TIDE) algorithms can be used to predict the sensitivity of glioma patients to immunotherapy. The TIDE score is negatively correlated with the sensitivity to immunotherapy (Jiang et al., 2018). The sensitivity of patients in the HR and LR groups to common chemotherapy drugs for gliomas was predicted through the R software package “pRRophetic”. The sensitivity is reflected by the IC50 of the drug which has a negative correlation with drug sensitivity.

### Nomogram

The R software package “rms” was used for integrating the records of survival time, survival status, SIRT-based signature, age, gender, pathological grade, and IDH status. A nomogram was established using a Cox test to evaluate the prognostic significance of these characteristics in TCGA samples, and the efficacy of the nomogram was evaluated using calibration and ROC curves.

### qPCR

Given the importance of SIRT1 and SIRT5 in glioma, we verified their expression using qPCR. RNAiso Plus (1 ml; Takara Bio, Japan) was used to isolate RNA, which was then reverse-transcribed using a PrimeScript™ RT reagent Kit (Takara Bio) according to the manufacturer’s instructions. qPCR was performed to quantify RNA levels using Hieff™ qPCR SYBR Green Master Mix (Yeasen Biotechnology, China) and the primers listed in Supplementary Material S1. ACTB was used as a standardized control.

### Western blot

Total protein was isolated using RIPA lysis buffer (Servicebio, China). The protein with the corresponding volume of loading buffer was boiled in a water bath for 10 min, after which the isolated proteins were separated through SDS-PAGE, transferred to a PVDF membrane, and incubated with primary and secondary antibodies including SIRT1 (Abclonal, A17307), SIRT5 (Proteintech, 15122-1-AP), and actin (Abclonal, AC038).

### Statistical analyses

To test differences in continuous variables between the two groups, *t*-tests or Mann–Whitney *U*-tests were used, depending on the distribution of the data. Continuous variables were compared among the three groups of samples using Kruskal Wallis tests. Chi-squared tests were applied to classify the data. All correlation analyses were performed using a Spearman correlation test. Kaplan–Meier survival analysis and the logrank method were applied to evaluate survival rates. All statistical analyses were performed using R software; statistical significance is reported at *p* < 0.05.

## Results

### Upregulated SIRT family genes in glioma

**FIGURE 1:**
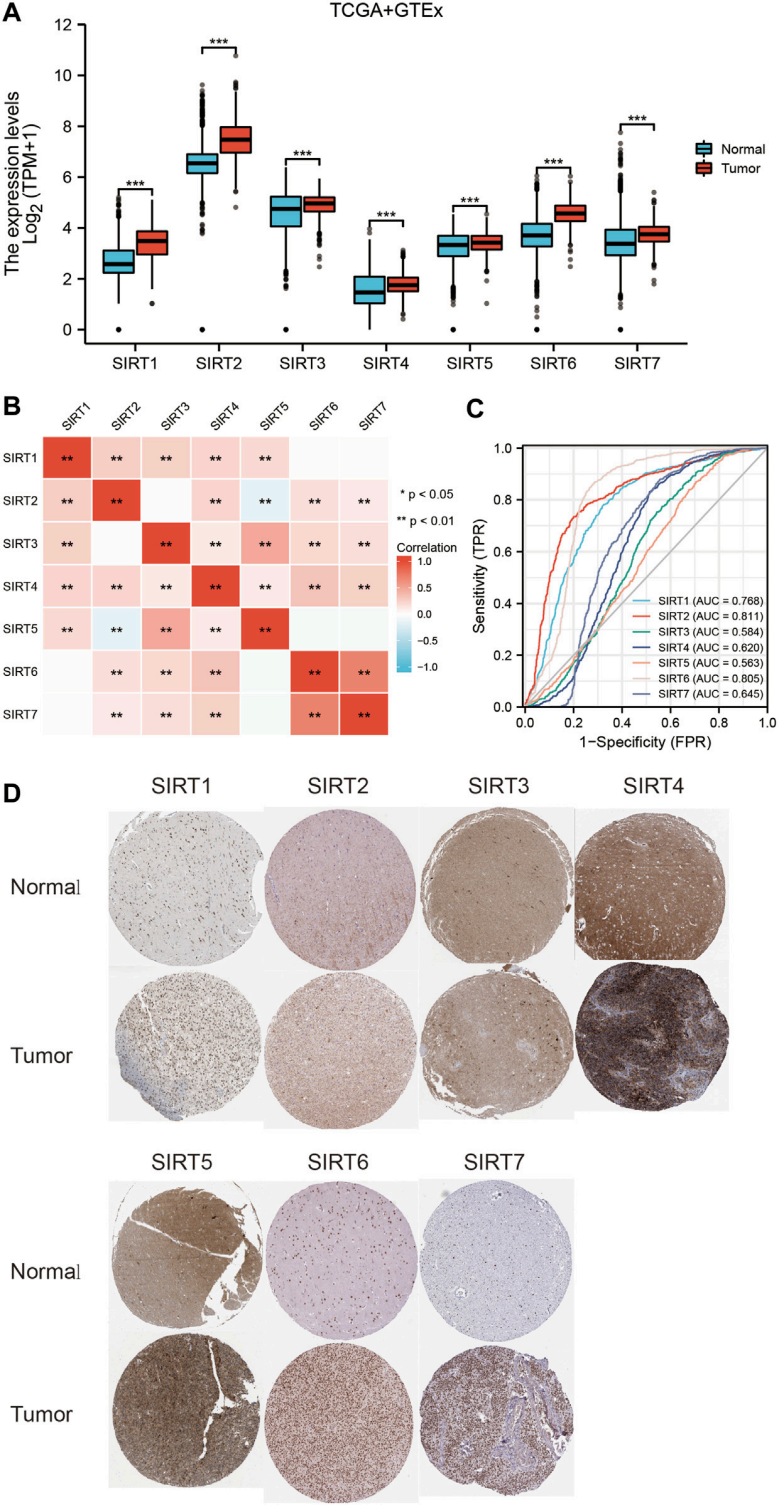
Expression levels and diagnostic values of SIRT genes in gliomas. **(A)** Differential expression of SIRT genes between glioma and normal brain tissues in TCGA and GETx databases. **(B)** Correlation analysis of SIRT gene expression in glioma tissues of the TCGA database. **(C)** Diagnostic ROC curves of SIRT genes in TCGA and GETx databases. **(D)** Expression levels of SIRT genes in glioma vs. normal brain tissues of HPA database (*p < 0.05, **p < 0.01, ***p < 0.001, ns: *p* > 0.05).

The joint TCGA and GTEx analysis displayed that at the transcriptional level, expression levels of SIRT genes were significantly higher in glioma samples than in normal brain tissues (Figure 1A). Figure 1B shows the correlation between SIRT gene expression in TCGA gliomas. The ROC curve tests showed that SIRT2 (AUC = 0.811) and SIRT6 (AUC = 0.805) can effectively distinguish glioma samples from normal brain tissue samples (Figure 1C). Figure 1D shows SIRT gene expression in gliomas and normal brain tissues in the HPA database. The results confirmed significantly higher expression of SIRT family genes in gliomas.

### Genetic changes of SIRT genes in gliomas

**FIGURE 2:**
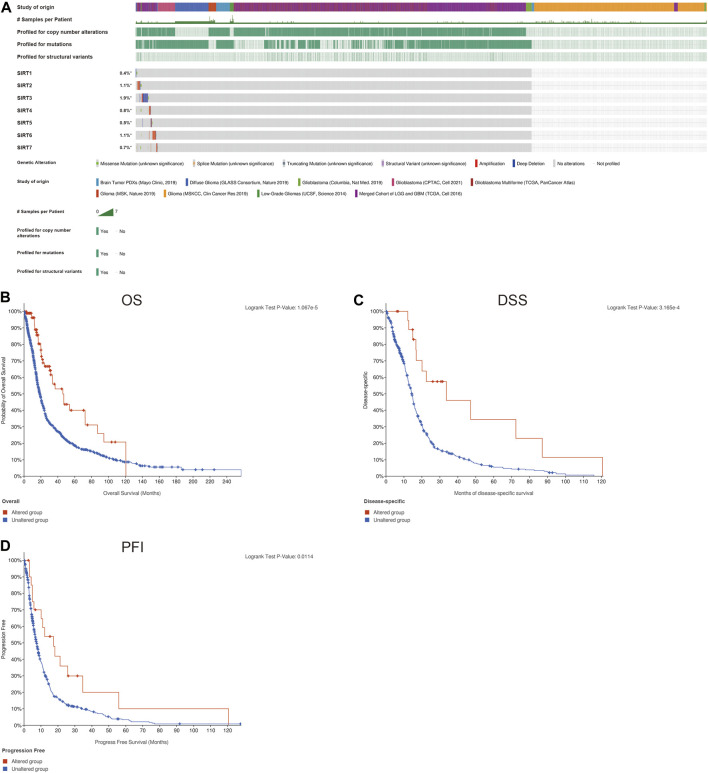
Genetic changes of SIRT family members in gliomas. **(A)** Mutations in SIRT family members in gliomas. **(B)** The association of SIRT gene mutations and OS in glioma patients. **(C)** Association of SIRT gene mutations and DSS in glioma patients. **(D)** Association of SIRT gene mutations and PFI in glioma patients (*p < 0.05, **p < 0.01, ***p < 0.001, ns: *p* > 0.05).

The genetic changes of SIRT genes in gliomas were explored, and the highest mutation rate occurred in SIRT3 with 1.9%, and the main form of mutation form was deletion (Figure 2A). The mutation frequencies of SIRT2 and SIRT6 were somewhat lower, with 1.1%, each, and mainly amplification mutations (Figure 2A). In addition, we also found a significant correlation between the gene mutations in SIRT family members and the prognosis of glioma patients as patients without mutations had worse OS, DSS, and PFI, in comparison with those bearing mutations in SIRT genes (Figures 2B–D) (*p* < 0.05).

### SIRT family genes are related to the prognosis of glioma

**FIGURE 3:**
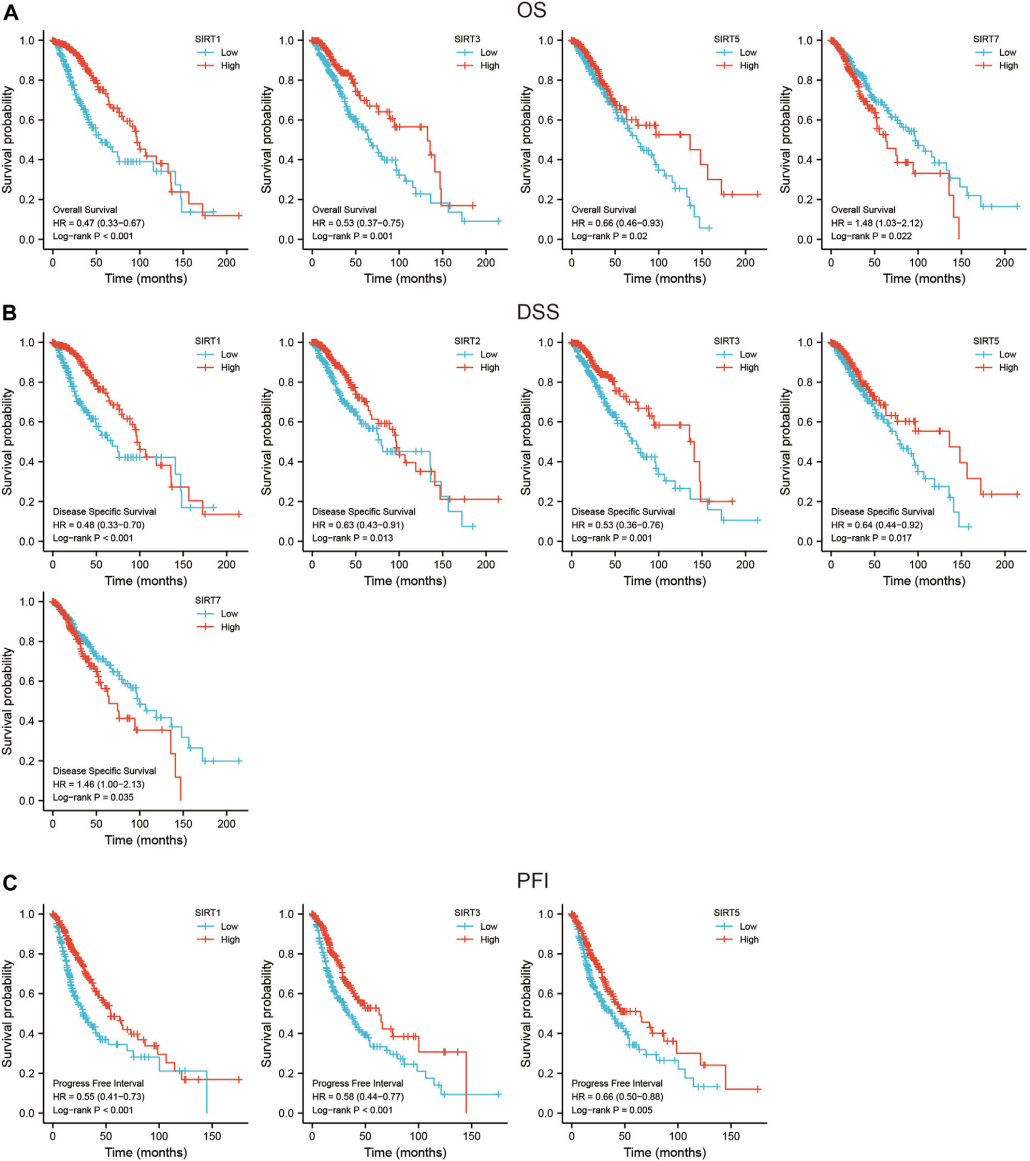
Prognostic value of SIRT genes in glioma. **(A)** SIRT family members associated with OS in glioma patients. **(B)** SIRT family members associated with DSS in glioma patients. **(C)** SIRT family members associated with PFI in glioma patients (*p < 0.05, **p < 0.01, ***p < 0.001, ns: *p* > 0.05).

The SIRT family was grouped based according to the median expression value of each gene to evaluate their prognostic value in the TCGA data set. The evaluation indicators included OS, DSS, and PFI. The results showed that SIRT1, SIRT3, and SIRT5 were associated with better OS, while SIRT7 was associated with worse OS (Figure 3A) (*p* < 0.05). SIRT1, SIRT2, SIRT3, and SIRT5 were associated with better DSS, while SIRT7 was associated with worse DSS (Figure 3B) (*p* < 0.05). SIRT1, SIRT2, and SIRT3 were associated with better PFI (Figure 3C) (*p* < 0.05).

To evaluate whether SIRT family members had independent predictive ability for the prognosis of glioma patients, we included SIRT gene expression and clinical information of TCGA glioma patients such as gender, age, pathological grade, and IDH status in univariate and multivariate Cox analyses. The results (Table 1) suggest high expression of SIRT1 as an independent prognostic factor which can predict OS in glioma patients (*p* < 0.05). Tables 2, 3 show that the high expression of SIRT5 is a good independent predictor of DSS and PFI, respectively, in glioma patients (*p* < 0.05), whereas, high expression of SIRT7 was an independent predictor of poor PFI in glioma patients (*p* < 0.05).

**TABLE 1:** SIRT family and clinical characteristics as analyzed by OS-based univariate and multivariate Cox regression in the TCGA database.

Characteristics	Total(N)	Univariate analysis		Multivariate analysis	
		Hazard ratio (95% CI)	*p*-value	Hazard ratio (95% CI)	*p*-value
Gender	669				
Female	283	Reference			
Male	386	1.230 (0.955–1.585)	0.109	1.081 (0.819–1.426)	0.584
WHO grade	612	19.164 (12.573–29.209)	<0.001	3.144 (1.710–5.779)	<0.001
IDH status	660				
WT	237	Reference			
Mut	423	0.102 (0.077–0.135)	<0.001	0.352 (0.217–0.573)	<0.001
Age	669	1.066 (1.056–1.076)	<0.001	1.034 (1.021–1.047)	<0.001
SIRT1	669	0.189 (0.148–0.242)	<0.001	0.657 (0.444–0.971)	0.035
SIRT2	669	0.574 (0.477–0.690)	<0.001	0.982 (0.803–1.201)	0.862
SIRT3	669	0.346 (0.251–0.478)	<0.001	0.897 (0.581–1.384)	0.623
SIRT4	669	0.695 (0.485–0.996)	0.047	1.158 (0.753–1.781)	0.503
SIRT5	669	0.265 (0.170–0.412)	<0.001	0.572 (0.327–1.000)	0.050
SIRT6	669	1.288 (0.957–1.733)	0.095	0.655 (0.418–1.026)	0.064
SIRT7	669	1.880 (1.372–2.576)	<0.001	1.168 (0.717–1.904)	0.533

**TABLE 2:** The SIRT family and clinical features were analyzed by DSS-based univariate and multivariate Cox regression in the TCGA database.

Characteristics	Total(N)	Univariate analysis		Multivariate analysis	
		Hazard ratio (95% CI)	*p*-value	Hazard ratio (95% CI)	*p*-value
Gender	648				
Female	275	Reference			
Male	373	1.229 (0.940–1.607)	0.132	1.007 (0.745–1.361)	0.963
WHO grade	590	19.859 (12.672–31.123)	<0.001	3.086 (1.620–5.877)	<0.001
IDH status	639				
WT	223	Reference			
Mut	416	0.096 (0.071–0.130)	<0.001	0.309 (0.186–0.513)	<0.001
Age	648	1.066 (1.056–1.077)	<0.001	1.034 (1.021–1.048)	<0.001
SIRT1	648	0.189 (0.146–0.245)	<0.001	0.723 (0.475–1.102)	0.132
SIRT2	648	0.550 (0.451–0.670)	<0.001	0.964 (0.775–1.200)	0.745
SIRT3	648	0.330 (0.232–0.468)	<0.001	0.798 (0.490–1.302)	0.367
SIRT4	648	0.694 (0.474–1.017)	0.061	1.180 (0.749–1.858)	0.475
SIRT5	648	0.260 (0.163–0.415)	<0.001	0.533 (0.293–0.969)	0.039
SIRT6	648	1.206 (0.882–1.650)	0.240	0.642 (0.400–1.031)	0.067
SIRT7	648	1.823 (1.308–2.541)	<0.001	1.204 (0.724–2.003)	0.474

**TABLE 3:** SIRT family and clinical characteristics as analyzed by PFI-based univariate and multivariate Cox regression in the TCGA database.

Characteristics	Total(N)	Univariate analysis		Multivariate analysis	
		Hazard ratio (95% CI)	*p*-value	Hazard ratio (95% CI)	*p*-value
Gender	669				
Female	283	Reference			
Male	386	1.068 (0.854–1.336)	0.564	1.023 (0.806–1.297)	0.853
WHO grade	612	7.900 (5.766–10.822)	<0.001	1.584 (0.979–2.562)	0.061
IDH status	660				
WT	237	Reference			
Mut	423	0.140 (0.110–0.180)	<0.001	0.285 (0.182–0.445)	<0.001
Age	669	1.041 (1.033–1.049)	<0.001	1.013 (1.002–1.023)	0.015
SIRT1	669	0.255 (0.204–0.318)	<0.001	0.754 (0.532–1.068)	0.112
SIRT2	669	0.653 (0.552–0.771)	<0.001	1.014 (0.841–1.224)	0.881
SIRT3	669	0.415 (0.310–0.555)	<0.001	0.844 (0.572–1.245)	0.393
SIRT4	669	0.681 (0.490–0.947)	0.022	1.182 (0.796–1.757)	0.407
SIRT5	669	0.312 (0.213–0.457)	<0.001	0.519 (0.326–0.828)	0.006
SIRT6	669	1.106 (0.854–1.433)	0.443	0.590 (0.397–0.878)	0.009
SIRT7	669	1.630 (1.223–2.171)	<0.001	1.621 (1.035–2.540)	0.035

### PPI network and function enrichment analysis

**FIGURE 4:**
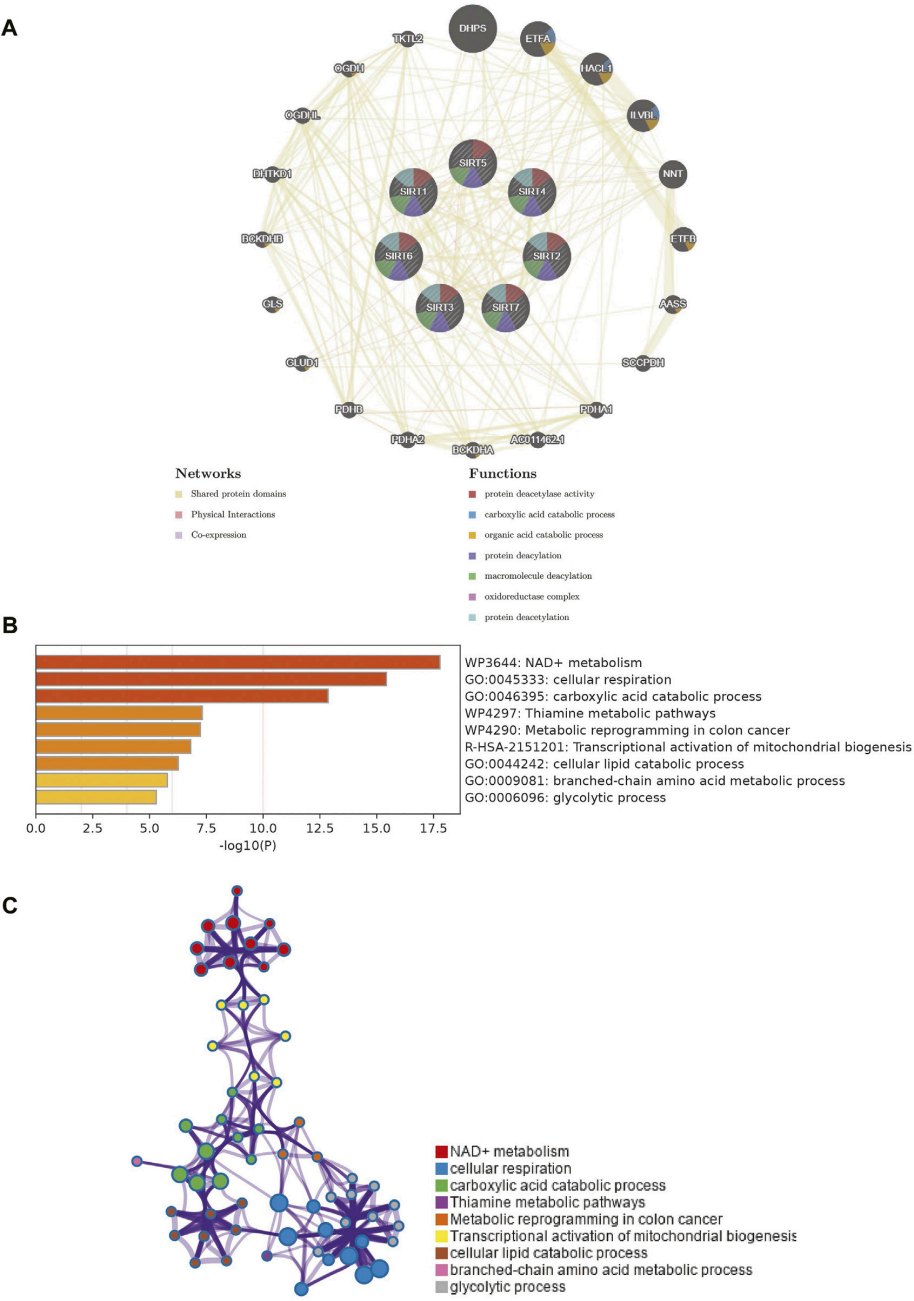
PPI network of SIRT family members and enrichment analysis of network genes. **(A)** PPI network of SIRT family members and their interacting proteins. **(B)** Gene enrichment in the PPI network as analyzed using Metascape. **(C)** Network diagram of enrichment analysis results (*p < 0.05, **p < 0.01, ***p < 0.001, ns: *p* > 0.05).

To identify the proteins that may interact with SIRT family genes, we developed and visualized a PPI network using the GeneMANIA database (Figure 4A). The results of GeneMANIA produced 20 proteins in interaction with the SIRT genes. DHPS, ETFA, HACL1, and ILVBL were the closest respective ones. Functioning of SIRT family genes and their related proteins are pivotally related to biological processes such as protein-protein deacetylase activity, carboxylic acid catabolism, organic acid catabolism, protein deacylation, macromolecule deacylation, oxidoreductase complex, and protein deacetylation (Figure 4A). To explore the possible biological processes involved in the SIRT gene family, we used the Metascape database to analyze functional enrichment of the SIRT family and interacting genes.

SIRT genes are mainly enriched in biological processes such as NAD + metabolism, cellular respiration, carboxylic acid catabolic process, thiamine metabolic pathways, metabolic reprogramming in colon cancer, transcriptional activation of mitochondrial biogenesis, cellular lipid catabolic process, branched-chain amino acid metabolic process, and glycolytic process (Figure 4B). Figure 4C shows the interaction network of these biological processes.

### Establishing and verifying a SIRT-based signature

**FIGURE 5:**
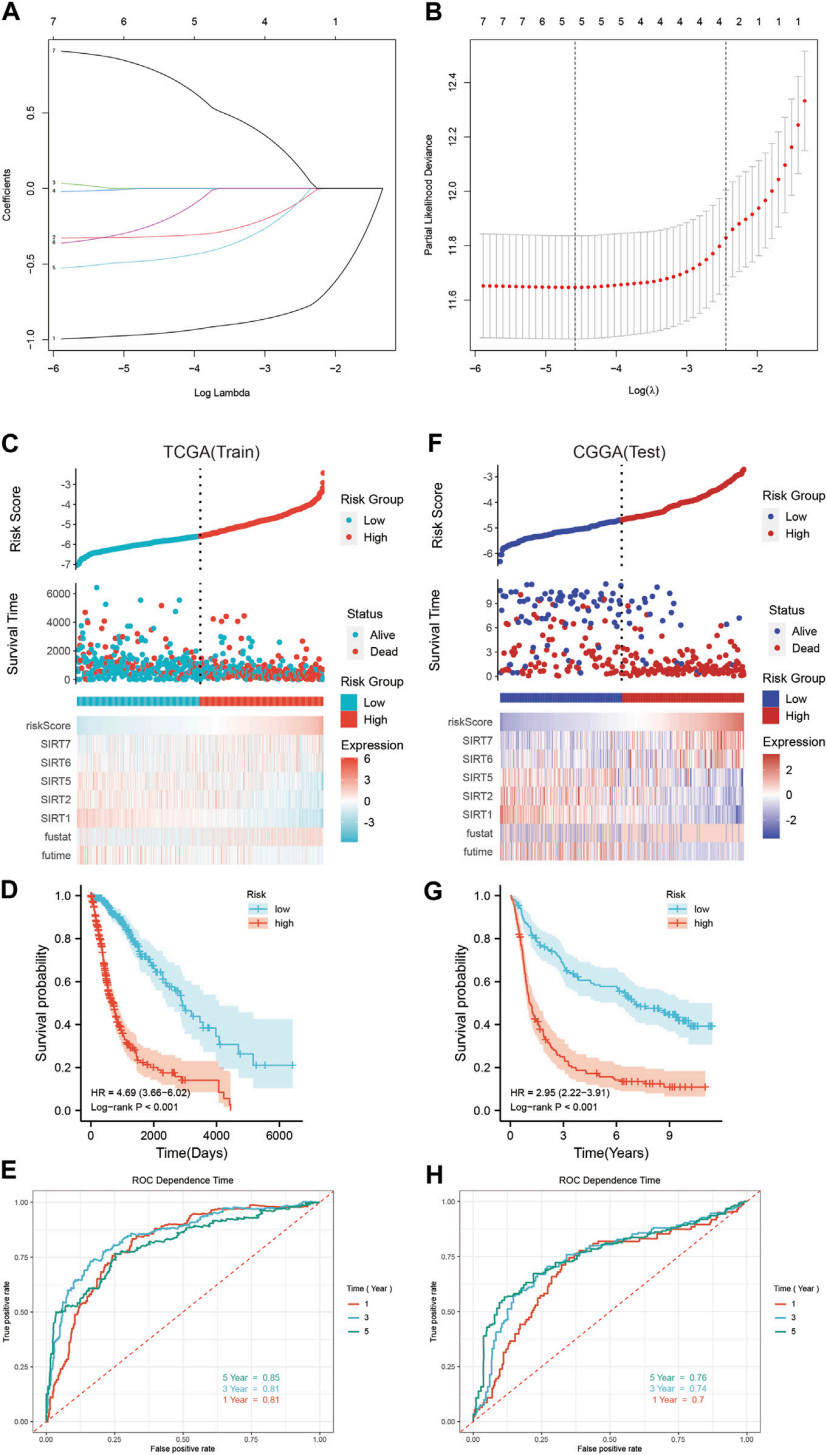
Construction and verification of SIRT-based signature. **(A)** Lasso coefficients of SIRT gene. **(B)** Relationship between deviance and log(lambda). **(C)** The distribution of risk scores, survival time and status, as well as the five SIRT genes’ expression profile in glioma patients of the TCGA database. **(D)** Kaplan–Meier survival analysis of OS in TCGA database. **(E)** The SIRT-based signature predicts the time-dependent ROC curves of 1 -, 3 -, and 5-year OS in glioma patients in the TCGA database. **(F)** Distribution of risk scores, survival time and status, and the five SIRT gene expression profiles in glioma patients of the CGGA database. **(G)** Kaplan–Meier survival analysis of OS in the CGGA database. **(H)** SIRT-based signature predicts the time-dependent ROC curves of 1 -, 3 -, and 5-year OS in glioma patients in the CGGA database (*p < 0.05, **p < 0.01, ***p < 0.001, ns: *p* > 0.05).

Using the TCGA database, the SIRT genes were analyzed by LASSO regression tests followed by multivariate Cox regression (Figures 5A,B), based on which SIRT-based signatures containing the five SIRT family genes SIRT1, SIRT2, SIRT5, SIRT6, and SIRT7 were generated. The expression levels of these five genes and their coefficients in a linear combination were used to determine the risk score for each sample, as follows:

$$Risk\;score=SIRT1\times \left(-0.961\right)+SIRT2\times \left(-0.318\right)+SIRT5\times \left(-0.473\right)+SIRT6\times \left(-0.239\right)+SIRT7\times \left(0.784\right)$$

Accordingly, the risk score was calculated for every glioma patient in the TCGA database which was used for stratifying the patients in HR and LR groups according to the median risk score (Figure 5C). Figure 5C illustrates the survival status and survival time of the HR group and LR patients. The scatter chart shows the higher mortality in HR patients. The expression profiles of these five SIRT genes in the HR and LR groups (TCGA database) are shown in Figure 5C. According to the outcomes of Kaplan–Meier survival analyses, OS was significantly lower in the HR than in the LR group (Figure 5D). In addition, time-dependent ROC curve analyses showed that the AUC values of 1-, 3-, and 5-year OS were 0.81, 0.81, and 0.85, respectively (Figure 5E), which indicates the potential of a SIRT-based signature for accurate prediction of OS in glioma patients.

The CGGA queue was used as a test dataset for further verification of accuracy and universality of the SIRT-based signature. Then, the risk score of patients in this queue was calculated using the risk score formula obtained in the train dataset, and patients were divided into the HR and LR groups according to the median value (Figure 5F). The survival status and survival time of the patients from the CGGA database and the expression patterns of the five SIRT genes are shown in Figure 5F. Kaplan–Meier survival analysis showed higher OS in LR than in HR patients (Figure 5G). Time-dependent ROC curve analysis indicated 1-, 3-, and 5-year OS AUC values of 0.7, 0.74, and 0.76, respectively, which indicates that the SIRT-based signature is accurate and specific for predicting OS of glioma patients (Figure 5H).

### The SIRT-based signature can independently predict OS of glioma patients

Univariate and multivariate Cox regression analyses were used to determine the potential of the SIRT-based signature of independently predicting OS in glioma patients as well as their clinical features in TCGA and CGGA databases. In the TCGA database, SIRT-based signature, age, pathological grade, and the IDH status were independent predictors (Table 4). In the CGGA database, SIRT-Based signature, pathological grade and 1p19q co-deletion were independent predictors (Table 5).

**TABLE 4:** The SIRT-Based signature and clinical features were analyzed by OS-based univariate and multivariate Cox regression in the TCGA database.

Characteristics	Total(N)	Univariate analysis		Multivariate analysis	
		Hazard ratio (95% CI)	*p*-value	Hazard ratio (95% CI)	*p*-value
Riskscore	686	2.882 (2.495–3.330)	<0.001	1.559 (1.177–2.066)	0.002
Age	686	1.066 (1.057–1.076)	<0.001	1.049 (1.033–1.064)	<0.001
Gender	686				
Male	395	Reference			
Female	291	0.818 (0.638–1.049)	0.114	0.808 (0.560–1.166)	0.255
Histological Grade	612	3.407 (2.324–4.994)	<0.001	2.123 (1.414–3.187)	<0.001
IDH Mutation	672				
WT	242	Reference			
Mutant	430	0.108 (0.082–0.142)	<0.001	0.343 (0.219–0.539)	<0.001

**TABLE 5:** The SIRT-Based signature and clinical features were analyzed by OS-based univariate and multivariate Cox regression in the TCGA database.

Characteristics	Total(N)	Univariate analysis		Multivariate analysis	
		Hazard ratio (95% CI)	*p*-value	Hazard ratio (95% CI)	*p*-value
riskScore	302	2.069 (1.737–2.466)	<0.001	1.255 (0.998–1.579)	0.050
Grade	302	7.957 (5.334–11.871)	<0.001	5.291 (3.416–8.195)	<0.001
Gender	302				
Male	186	Reference			
Female	116	1.083 (0.819–1.431)	0.576	0.969 (0.729–1.289)	0.829
Age	302	1.031 (1.018–1.044)	<0.001	1.012 (0.998–1.025)	0.085
IDH Mutation	302				
Wildtype	138	Reference			
Mutant	164	0.384 (0.289–0.509)	<0.001	1.391 (0.946–2.046)	0.093
1p19q codeletion	302				
Non-codel	239	Reference			
Codel	63	0.168 (0.102–0.277)	<0.001	0.258 (0.150–0.442)	<0.001

### The SIRT-based signature can predict DSS and PFI in glioma patients

**FIGURE 6:**
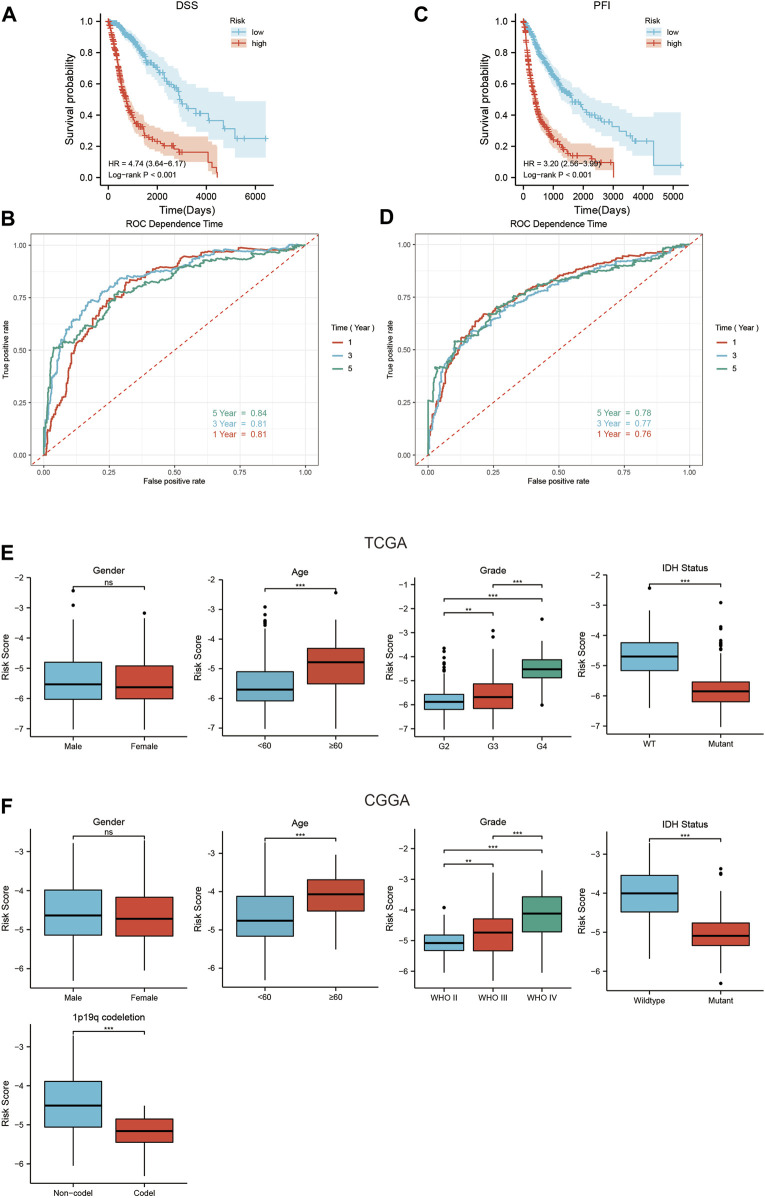
The relationship between the SIRT-Based signature and DSS, PFI, and clinical characteristics of glioma patients. **(A)** The SIRT-based signature predicts the Kaplan–Meier survival of DSS in the TCGA queue. **(B)** The SIRT-based signature predicts the time-dependent ROC curves of 1-, 3-, and 5-year DSS of glioma patients in the TCGA database. **(C)** The SIRT-Based signature predicts the Kaplan-Meier survival of PFI in the TCGA queue. **(D)** The SIRT-based signature predicts the time-dependent ROC curves of 1-, 3-, and 5-year PFI of glioma patients in the TCGA database. **(E)** Relationship between SIRT-Based signature and sex, age, pathological grade, and IDH status of glioma patients in TCGA database. **(F)** Relationship between the SIRT-based signature and sex, age, pathological grade, IDH status, and 1p19q co-deletion of glioma patients in the CGGA database (*p < 0.05, **p < 0.01, ***p < 0.001, ns: *p* > 0.05).

The TCGA database included DSS and PFI information of glioma patients, and we assessed whether the SIRT-based signature can predict DSS and PFI in TCGA queues. The results showed that the DSS and PFI in LR patients were higher than those in HR patients (Figures 6A,C). The time-dependent ROC curve analysis indicated that after 1, 3, and 5 years, the AUC values of DSS were 0.81, 0.81, and 0.84, respectively (Figure 6B), and those of PFI were 0.7, 0.74, and 0.76, respectively (Figure 6D). To explore the relationship between SIRT-based signature and clinical characteristics, we performed a correlation analysis between the risk scores and clinical characteristics of patients in the TCGA and CGGA databases. In the TCGA database, patients with advanced age, high pathological grade, and IDH wild-type glioma tended to show higher risk scores (Figure 6E). In the CGGA database, patients with advanced age, high pathological grade, IDH wild type, and no 1p19q co-deletion glioma tended to have high risk scores (Figure 6F).

### The SIRT-based signature can evaluate immune cell infiltration and immune status

**FIGURE 7:**
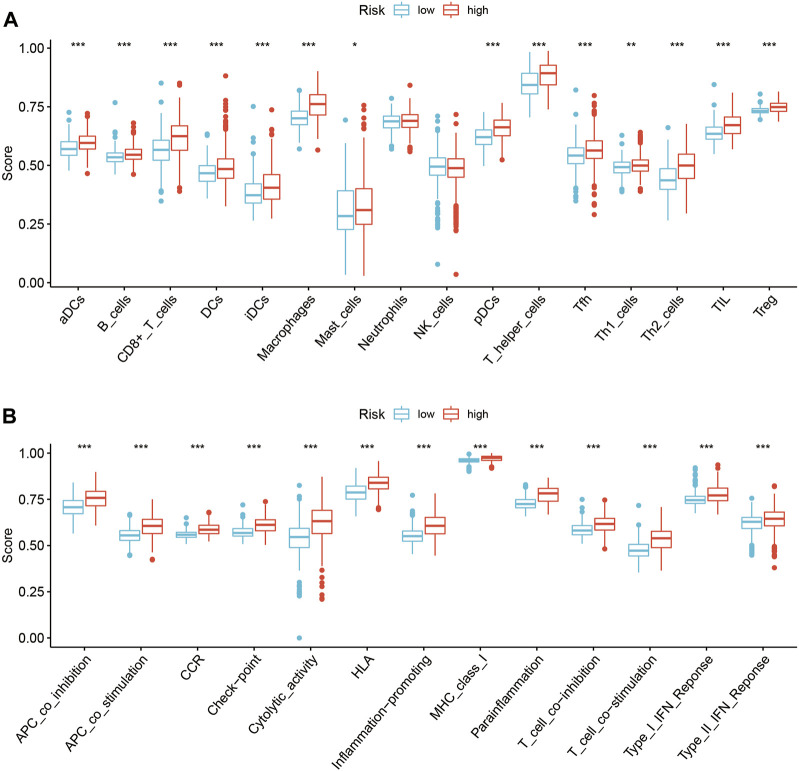
Relationship between the SIRT-based signature and immune characteristics of glioma patients. **(A)** The ssGSEA algorithm was used to calculate the difference in immune cell infiltration between HR and LR glioma patients in the TCGA database. **(B)**The ssGSEA algorithm was used to calculate the difference in immune status scores between high and LR groups in the TCGA database (*p < 0.05, **p < 0.01, ***p < 0.001, ns: *p* > 0.05).

The types of infiltrating immune cells in all TCGA glioma samples in the TCGA database were evaluated using ssGSEA algorithms (Figure 7A). The scores of several immune subgroups such as DCs, aDCs, iDCs, pDCs, B cells, CD8^+^ T cells, macrophages, mast cells, T helper cells, Tfh, Th1 cells, Th2 cells, and TIL were higher in the HR than in the LR group. The scores of several immune processes including APC co-inhibition, APC co-stimulation, CCR, check-point, cytolytic activity, HLA, inflammation-promoting, MHC class I, para-inflammation, T cell co-inhibition, T cell co-stimulation, type I IFN response, and type II IFN response were higher in HR than in LR patients (Figure 7B). These findings suggest that the SIRT-based signature reflects infiltration by various immune cells and the glioma patients’ immune status.

### Using the SIRT-based signature to evaluate the sensitivity of glioma patients to drug therapy

**FIGURE 8:**
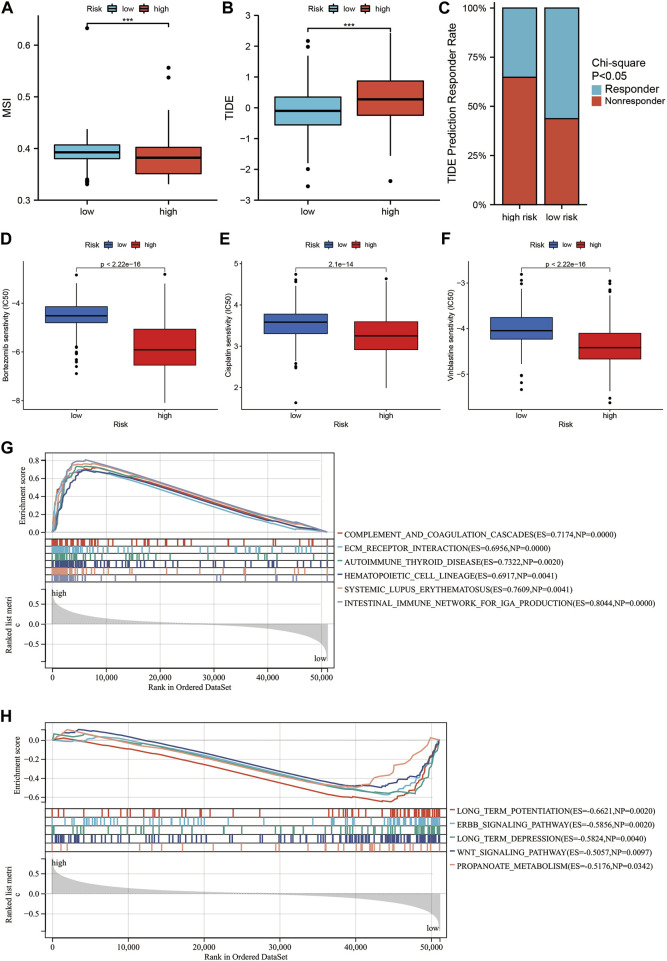
Relationship between the SIRT-based signature and drug sensitivity in glioma patients and GSEA. **(A)** Differences in MSI scores between HR and LR glioma patients in the TCGA database. **(B)** The difference in TIDE score between HR and LR glioma patients in the TCGA database. **(C)** Comparison of the percentage of responders to immunotherapy among high and LR glioma patients in the TCGA database. **(D–F)** Comparison of sensitivity to Bortezomib **(D)**, Vinblastine **(E)**, and Cisplatin **(F)** in high and LR glioma patients in TCGA database. **(G,H)** Top5 KEGG pathways were significantly enriched in the TCGA database in the HR group **(G)** and LR group **(H)** (*p < 0.05, **p < 0.01, ***p < 0.001, ns: *p* > 0.05).

In recent years, immune checkpoint inhibitors (ICIs) have emerged as a milestone during the attempts of treating glioma (Tian et al., 2022). However, only few patients benefit from it, and most patients show no response to ICI treatment. Thus, the current study examined the relationship between SIRT-based signature and sensitivity to the ICI treatment. We found that HR patients had lower MSI and higher TIDE scores than LR patients (Figures 8A,B). The TIDE algorithm can predict whether patients will respond to ICI treatment. Accordingly, the HR glioma patients showed a lower rate of sensitivity in response to ICI treatment compared to LR patients (*p* < 0.05) (Figure 8C).

Subsequently, we explored difference in the sensitivity to treatment with common chemotherapy drugs between HR and LR glioma patients using the “pRRophetic” software package. The results showed a significantly lower IC50 for Bortezomib, Vinblastine, and Cisplatin in the HR than in the LR group (Figures 8D–F). This indicates that HR glioma patients showing higher sensitivity can benefit more from a combination therapy with Bortezomib, Vinblastine, and Cisplatin.

### Functions related to the SIRT-based signature

To compare the immune-related pathways between patients from HR and LR groups, GSEA analysis was conducted, which showed a significant enrichment in immune-related pathways such as complement and coagulation cascades, ECM receptor interaction, and intestinal immune network for IGA production in HR patients (Figure 8G). By contrast, in LR patients, a significant enrichment occurred in some signal pathways such as long term potentiation, ERBB signaling pathway, long-term depression, Wnt signaling pathway, and propanoate metabolism (Figure 8H).

### Construction of a nomogram

**FIGURE 9:**
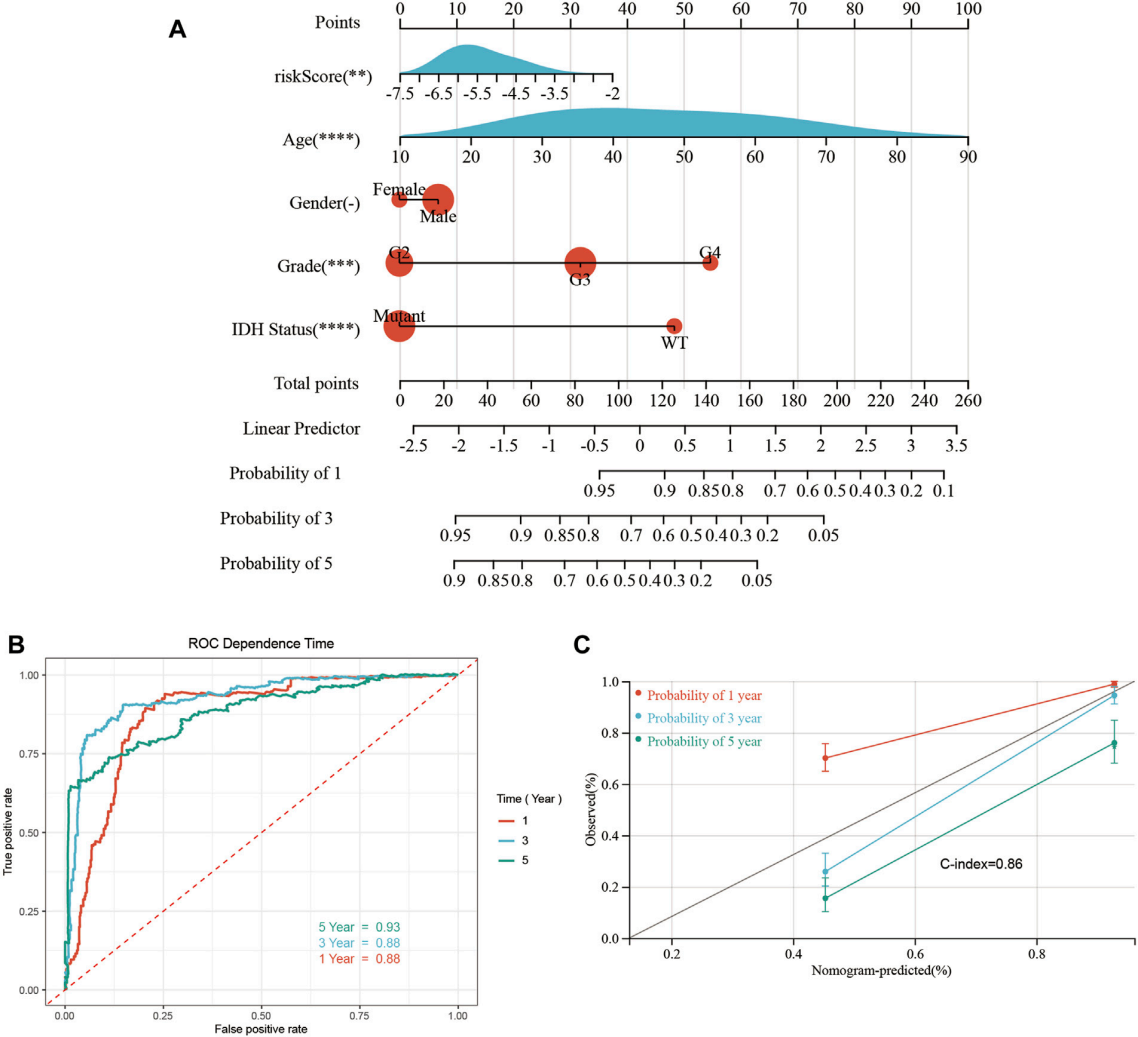
Construction and performance test of the nomogram. **(A)** Nomograms for predicting 1-, 3-, and 5-year OS in glioma patients. **(B)** Time-dependent ROC curves analysis was used to evaluate the OS of glioma patients at 1, 3, and 5 years. **(C)** The calibration curve of OS in glioma patients at 1-, 3-, and 5-year stages was evaluated through a nomogram (*p < 0.05, **p < 0.01, ***p < 0.001, ns: *p* > 0.05).

A predictive quantitative nomogram was developed to evaluate the clinical prognosis of glioma patients. Our OS nomogram included gender, age, risk scores, pathological grades, and IDH status records as per the TCGA database (Figure 9A). Time-dependent ROC curve analysis displayed AUC values of 0.88, 0.88, and 0.93 for 1-, 3-, and 5-year OS, respectively (Figure 9B). Calibration of the curve showed high consistency between the nomogram prediction and the actual results, with a C-index of 0.86 (Figure 9C). This confirms that nomograms have a high predictive potential for OS in patients with glioma.

### Verifying expression of SIRT genes

**FIGURE 10:**
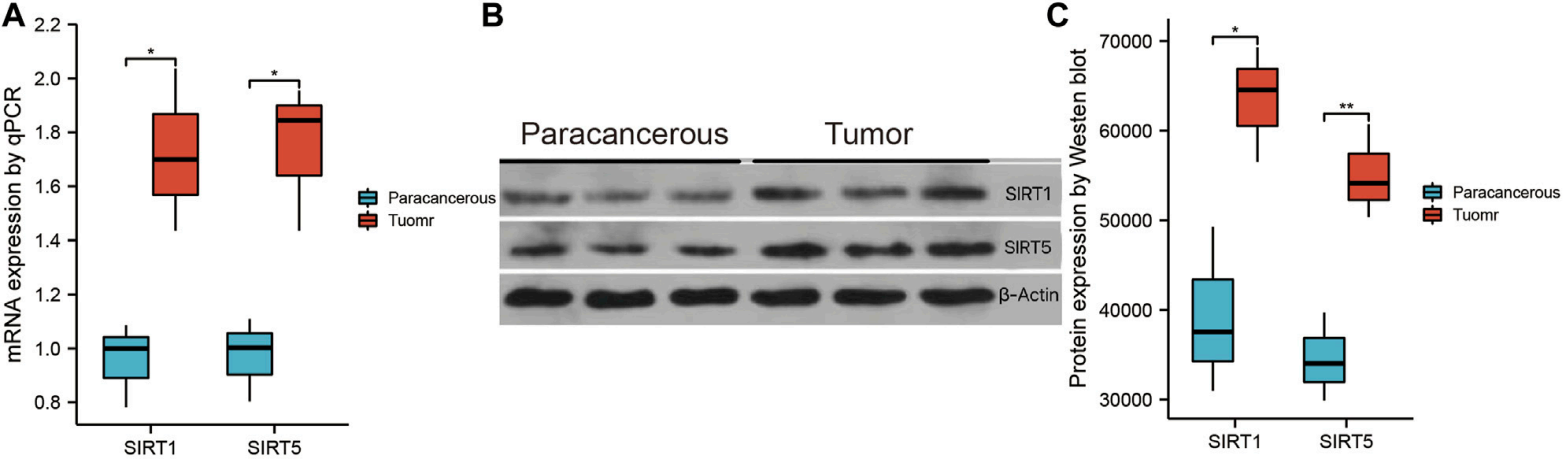
Validation of SIRT1 and SIRT5 expression. **(A)** qPCR was used to detect the differential expression of SIRT1 and SIRT5 between glioma and paracancerous tissue. **(B)** Western blotting was used to detect differential expression of SIRT1 and SIRT5 between glioma and paracancerous tissue (*p < 0.05, **p < 0.01, ***p < 0.001, ns: *p* > 0.05).

Subsequently, we verified the differential expression of SIRT1 and SIRT5 between GBM tissues and paracancerous tissues by western blotting and qPCR. The qPCR results showed that the expression of SIRT1 and SIRT5 was higher in GBM tissues than in paracancerous tissues (Figure 10A). Consistent results were obtained through western blotting (Figures 10B,C).

## Discussion

High-throughput sequencing approaches including RNS sequencing and transcriptomics, and the discovery of tumor-related genetic abnormalities have been increasingly used in cancer research and for diagnostic purposes (Hamilton et al., 2022; Iwamura et al., 2022). Some transcriptome records have been uploaded to open databases and are used for reanalysis to discover prognostic indicators and therapeutic targets of various malignant tumors (Elmallah et al., 2022; Iwamura et al., 2022). In recent years, research on the molecular characteristics of gliomas has put forward many potential markers, which can be used to classify gliomas, to judge prognosis, and to guide treatment (Guo et al., 2021). However, in isolation, these are insufficient for predicting the course of complex gliomas and lack the ability to determine effective treatment. In the current study, we developed a concise SIRT-based signature to characterize the prognosis of glioma. As expected, the SIRT-based signature was strongly associated with clinicopathological parameters indicating a poor prognosis of glioma. It is noteworthy that the SIRT-based signature is a powerful prognostic biomarker for gliomas (especially high-grade gliomas), and it facilitates identifying ICI responders. In conclusion, our work provides valuable information for the diagnosis, prognosis, and classification of glioma, and it may help optimize immunotherapy.

Various proteins are regulated by lysine acetylation, and sirtuins are considered a principal regulator contributing to various cell functions, including gene expression, DNA repair, telomerase activity, epithelial to mesenchymal transition, cell cycling, differentiation, apoptosis, proliferation, ageing, metabolism, and oxidative stress responses (O'Callaghan and Vassilopoulos, 2017). Many of these cellular functions are critical in tumor cell proliferation and differentiation. Sirtuin protein plays different roles in promoting or inhibiting cancer in glioma cases (Funato et al., 2018; Liu et al., 2022). The roles of SIRT1, SIRT2, SIRT3, and SIRT7 in glioma are not comprehensively understood. Several studies found that SIRT1 exerts anti-cancer effects in gliomas (Ye et al., 2019; Fang et al., 2020). Fang et al. (2020) showed that glioma patients with high SIRT1 expression had a better prognosis, and our study produced consistent results. This study also found that SIRT1 could inhibit the progression of glioma, while Circ_0005075 promoted the progression of glioma by inhibiting the expression of SIRT1 (Fang et al., 2020). In addition, SIRT1 activator SRT2183 inhibits the growth of glioma cells through endoplasmic reticulum stress pathway (Ye et al., 2019). Other studies foundevidence also shows that SIRT1 can promote the development of gliomas (Li et al., 2016; Huo et al., 2017; Lei et al., 2019). Lei et al. (2019) showed that Circular RNA hsa_circ_0076248 up-regulates the expression of SIRT1 by competitively binding to SIRT1 miR-181a, thus promoting the progression of glioma. Betulinic acid derivative B10 aggravates glioma by inhibiting the acetylation of SIRT1 (Huo et al., 2017). The role of SIRT2 in glioma is controversial (Zhang et al., 2020). A study showed that SIRT3 can be regulated by myricetin nanoliposomes and accelerate glioma cell apoptosis (Wang et al., 2018). However, a different study confirmed that the interaction between TRAP1 and SIRT3 can maintain stem cell viability of glioma cells (Park et al., 2019). SIRT7 promotes proliferation and invasion in glioma cells by activating the ERK/STAT3 signaling pathway (Mu et al., 2019). Furthermore, there is evidence that SIRT7 is positively regulated by MEG3 and plays a role in inhibiting glioma (Xu et al., 2018).

Chen et al. (2019) showed that SIRT5 is associated with better prognosis of glioma cases, which consistent with our results. However, the function of SIRT5 in glioma remains unknown. Current research supports the role of SIRT5 as a tumor suppressor in many cancers, and this effect is achieved by regulating metabolism-related pathways. SIRT5 can regulate bile acid metabolism and inhibit immune escape of liver cancer cells (Sun et al., 2022). In renal cell carcinoma, SIRT5 inhibits tumor progression through Warburg efect (Yihan et al., 2021). The loss of SIRT5 regulates Glutamine Metabolism and Glutathione Metabolism and Pyrimidine Metabolism promotes pancreatic cancer (Hu et al., 2021). The current study confirms the carcinogenic effects of SIRT6 in glioma. There is evidence that SIRT6 is regulated by MST1 and miR-33a and can inhibit the activity of glioma cells (Chang et al., 2017; Zhu et al., 2019). In conclusion, these findings suggest that sirtuin protein is related to glioma onset and progression, suggesting these proteins as potential biomarkers for evaluating prognosis of the glioma.

In conclusion, given their important role in gliomas, we performed a comprehensive analysis of seven members of the SIRT family in gliomas. All seven SIRT family genes showed significant up-regulation in glioma tissues, further indicating the critical role of SIRT in gliomas. In addition, the Kaplan–Meier curve showed that SIRT1, SIRT3, and SIRT5 were closely associated with OS, DSS, and PFI of glioma patients, and other SIRT family members are related to the prognosis of glioma patients to varying degrees. We thus established a SIRT-based signature and further divided the subjects into HR and LR groups. The Kaplan–Meier curve analysis of the train and test datasets indicated lower OS in HR than in LR patients. The time-dependent ROC curve test indicated the powerful prediction potential of the SIRT-based signature. Further, univariate and multivariate Cox analyses confirmed the independent prediction potential of the SIRT-based signature. It is worth mentioning that the SIRT-based signature shows a considerable potential for effectively predicting DSS and PFI in patients suffering from glioma. Our findings show that the established SIRT-based signature is accurate and clinically applicable for prognosis purposes in glioma patients. In addition, the current constructed and modified OS nomogram based on gender, age, risk scores, pathological grades, and IDH status showed a higher accuracy compared to nomograms based on a single risk score. Our nomogram is thus helpful for clinical decision-making and personalized management of glioma patients. SIRT family members have been studied in gliomas, however, there is considerable controversy, and the specific functions of these genes and proteins in glioma cases require further research. To the best of our knowledge, this is the first comprehensive and systematic study on the prognostic potential of SIRT family members in glioma, confirming the important role of this family and SIRT-based signature in glioma prognosis and providing valuable biomarkers for the prognostic management of this disease.

For further differentiating the biological functions between the HR and LR groups, we conducted GSEA analyses and discovered significant enrichment in immune-related biological processes in HR patients. The association between the SIRT-based signature and tumor immune cell infiltration was also evaluated, showing significant differences in the immune status and immune cell abundance between HR and LR patients. The importance of infiltrated immune cells for immunotherapy and prognosis has been recognized (Helmink et al., 2020), which suggests that responses to immunotherapy may differ between the two groups. MSI is a more effective marker for selecting glioma patients for immunotherapy (Mensali and Inderberg, 2022). Glioma patients with higher MSI scores are more sensitive to anti-PD-L1 treatment. The TIDE algorithm is an effective tool to predict whether cancer patients will respond to immunotherapy. Generally, a higher TIDE score indicates less sensitivity to immunotherapy (Jiang et al., 2018). However, the present results indicate that HR groups were less sensitive to immunotherapy, whether through MSI scores or the TIDE algorithm. In the present study, HR patients were more sensitive to the combination of Bortezomib, Vinblastine, and Cisplatin. According to previous studies, Bortezomib inhibits glioma growth and improves the efficacy of TMZ chemotherapy (Tang et al., 2019), while Vinblastine and Cisplatin were selected as they are commonly used chemotherapy drugs for glioma patients (Park et al., 2016; Rottenberg et al., 2021).

In conclusion, the present constructed SIRT-based signature of glioma showed that it is accurate for evaluating the prognosis of glioma patients and their sensitivity to drug treatment. Therefore, it provides a personalized scheme for the management of glioma patients. In addition, our SIRT-based signature showed a significant correlation with the tumor immune status and the infiltration level of different immune cells in the tumor microenvironment.

## Data Availability

The original contributions presented in the study are included in the article/Supplementary Material, further inquiries can be directed to the corresponding author.
